# Adhering to a national surgical care bundle reduces the risk of surgical site infections

**DOI:** 10.1371/journal.pone.0184200

**Published:** 2017-09-06

**Authors:** Mayke B. G. Koek, Titia E. M. Hopmans, Loes C. Soetens, Jan C. Wille, Suzanne E. Geerlings, Margreet C. Vos, Birgit H. B. van Benthem, Sabine C. de Greeff

**Affiliations:** 1 Department of Epidemiology and Surveillance, Centre for Infectious Disease Control (CIb), National Institute for Public Health and the Environment (RIVM), Bilthoven, the Netherlands; 2 Department of Medical Statistics, Leiden University Medical Centre, Leiden, the Netherlands; 3 Department of Infectious Diseases, Academic Medical Center, Amsterdam, the Netherlands; 4 Department of Medical Microbiology and Infectious Diseases, Erasmus MC, Rotterdam, the Netherlands; George Washington University, UNITED STATES

## Abstract

**Background:**

In 2008, a bundle of care to prevent Surgical Site Infections (SSIs) was introduced in the Netherlands. The bundle consisted of four elements: antibiotic prophylaxis according to local guidelines, no hair removal, normothermia and ‘hygiene discipline’ in the operating room (i.e. number of door movements). Dutch hospitals were advised to implement the bundle and to measure the outcome. This study’s goal was to assess how effective the bundle was in reducing SSI risk.

**Methods:**

Hospitals assessed whether their staff complied with each of the bundle elements and voluntary reported compliance data to the national SSI surveillance network (PREZIES). From PREZIES data, we selected data from 2009 to 2014 relating to 13 types of surgical procedures. We excluded surgeries with missing (non)compliance data, and calculated for each remaining surgery with reported (non)compliance data the level of compliance with the bundle (that is, being compliant with 0, 1, 2, 3, or 4 of the elements). Subsequently, we used this level of compliance to assess the effect of bundle compliance on the SSI risk, using multilevel logistic regression techniques.

**Results:**

217 489 surgeries were included, of which 62 486 surgeries (29%) had complete bundle reporting. Within this group, the SSI risk was significantly lower for surgeries with complete bundle compliance compared to surgeries with lower compliance levels. Odds ratios ranged from 0.63 to 0.86 (risk reduction of 14% to 37%), while a 13% risk reduction was demonstrated for each point increase in compliance-level. Sensitivity analysis indicated that due to analysing reported bundles only, we probably underestimated the total effect of implementing the bundle.

**Conclusions:**

This study demonstrated that adhering to a surgical care bundle significantly reduced the risk of SSIs. Reporting of and compliance with the bundle compliance can, however, still be improved. Therefore an even greater effect might be achieved.

## Introduction

In the last decade, much more attention has been paid internationally to Patient Safety and Infection Prevention. This was reflected in the Netherlands, where in 2007 a nationwide study was performed to quantify the amount of preventable complications and mortalities occurring in hospitals. This resulted in the formulation of a list of ten highly preventable complications, one of which was Surgical Site Infections (SSIs) [1]. SSIs are serious complications of surgical procedures and are associated with prolonged hospitalisation, re-interventions, morbidity or even death [2]. The risk of contracting an SSI can be reduced by performing care according to infection prevention guidelines, but literature shows that adherence to these guidelines is repeatedly low [3–6].

In 2008, following the results of the nationwide study, the Dutch Hospital Patient Safety Program (DHPSP) was set up to support Dutch hospitals by developing specific preventive programs, such as bundles of care. A bundle of care is a structured way of improving care and patient outcomes: a small set of three to five evidence-based practices which, when they are performed collectively and reliably, have been proven to improve patient outcomes [7, 8]. To develop an SSI bundle of care, the DHPSP appointed a multidisciplinary team of experts (incl. surgeons, anaesthesiologists, medical microbiologists and infection control practitioners) [9]. The experts selected four practices (elements) to be included in the SSI bundle of care (see Table 1): the appropriate administration of antibiotic prophylaxis (if indicated) according to local guidelines, no preoperative surgical site hair removal, perioperative normothermia, and exercising ‘hygiene discipline’ in the operating room. The first three elements were evidence-based, measurable and derived from the National Guidelines for the Prevention of SSIs [10–12]. Exercising hygiene discipline in the operating room was not evidence based, but nevertheless the expert team considered it ‘good practice’ and important to include in the bundle of care. However, as hygiene discipline by itself is difficult to measure, a maximum number of door openings during the surgery was designated as a surrogate marker for hygiene discipline. The allowed maximum number of door movements (‘agreed limit’) was not imposed by the DHPSP, but was to be determined by the hospitals.

**Table 1:** Four elements of the SSI-bundle.

Element	Definition	Explanatory remarks
**PAP-LG** (Peri-operative Antibiotic Prophylaxis according to Local Guidelines)	Did you comply with local guidelines for PAP? That is, if PAP was not indicated according to local guidelines, did you not administer PAP? OR If PAP was indicated according to local guidelines, did you: A) Follow the local guidelines for PAP concerning choice and dose of PAP (i.e. did you administer the appropriate PAP in the appropriate dose) AND B) Administer the PAP within 15–60 minutes before incision AND C) Repeat PAP if duration of operation > 4 hours or if major blood losses occur (i.e. > 2 litres)?	*Local guidelines*: *Based on national guidelines but choice of PAP and dose (item A) adapted to local situation*. *Answering options*: *Only one answer (‘Yes’ or ‘No’) possible*. *If according to guidelines PAP was not indicated*: *report ‘Yes’ if PAP was not given*. *If PAP was indicated*, *only report ‘Yes’ when complied with all three items (A+B+C*, *three part question)*
**(No) hair removal**	A) Did you not remove patient’s hair from the surgical site? OR B) If hair removal was necessary for surgical/technical reasons: did you remove the hair using a clipper?	*Report ‘Yes’ if you complied with item A or with item B*. *Other hair removing techniques are not allowed*.
**Peri-operative normothermia**	Did you ensure that patient’s body temperature at the end of the procedure was between 36–38°C (rectal measurement) or 35.5–37.5°C (non-rectal measurement)?	*Body temperature may be measured in recovery room*.
**Hygiene discipline** (Operating room door movements)	Did you keep the number of door openings of the operating room during the surgery (from start of incision to closure of skin) to an absolute minimum (i.e. below the agreed limit)?	*Hospitals determine a maximum number of door openings (limit X)*. *Limit may be determined for all surgeries combined or for each type of surgery separately*.

The surgical care bundle consisted of four elements. Compliance with each element could be answered with either Yes or No (one answer per element).

Dutch hospitals across the country were advised to implement the bundle when performing all types of surgeries and to measure the outcome in so called index surgeries (Table 2). The thirteen selected index surgeries were chosen by the DHPSP based on how often they are performed Dutch hospitals, and on their room for improvement in patient outcome. The DHPSP aimed to achieve a reduction in the incidence of SSIs and a 90% compliance with the total SSI prevention bundle [9].

**Table 2:** Index surgeries: Thirteen surgical procedures selected by the DHPSP.

Medical specialty	Procedure
Orthopaedic surgery (ORT)	total hip replacement
	total knee replacement
General surgery—breast (BRE)	mastectomy
General surgery—Gastro Intestinal (GIS)	laparoscopic cholecystectomy
	colon resection
Gynaecology (GYN)	abdominal hysterectomy
	vaginal hysterectomy
	Caesarean section
Neurosurgery (NEU)	laminectomy
Vascular surgery (VAS)	reconstruction of the abdominal aorta and abdominal vessels
Cardiovascular surgery (CAR)	Coronary Artery Bypass Grafting (CABG) heart valve replacement combined CABG and heart valve replacement

Compliance with the bundle is considered to contribute to a decreased SSI incidence, but it is also believed that the process of reporting and giving feedback, in itself, increases awareness among health care professionals of the significance of their role in patient safety and the importance of the prevention of SSIs [13]. In this paper, we retrospectively assess how effective implementing the DHPSP surgical care bundle was in reducing the risk of SSIs, by assessing the effect of bundle compliance on SSI risk. Additionally, we describe the reporting of (non-)compliance, and compliance with the bundle itself.

## Methods

### Design, definitions and data selection

A retrospective cohort study was performed using data available from the Dutch National Nosocomial Surveillance Network (PREZIES) [14]. Nearly all the hospitals in the Netherlands participate in this voluntarily surveillance and select one or several types of surgical procedures for surveillance on an annual basis. Briefly, PREZIES collects data about the patient, the surgery, a limited number of possible risk factors and the presence of an SSI. SSIs reported in the surveillance meet the criteria of the (European) Centre of Disease prevention and Control (CDC and ECDC) [15–18] and are confirmed by trained hospital personnel. Retrospective on-site validation was performed by the PREZIES-team. PREZIES distinguishes superficial SSIs from the deep SSIs, which include deep incisional SSIs and organ-space SSIs. SSIs in this study occurred, by definition, within 30 days after surgery (superficial and deep SSIs), or within one year after implant surgery (deep SSIs only) [15–18]. Detailed information on the national PREZIES surveillance has been described previously [14].

Within the SSI surveillance, from 2009 onwards PREZIES has been facilitating the (voluntary) reporting of (non-)compliance with the four SSI bundle elements, using the ‘Yes’ (compliant) or ‘No’ (non-compliant) answering possibilities as defined by the DHPSP [9]. Measuring and reporting of the bundle elements could be manual or (semi-)automated, and varied per element and per hospital. As reporting bundle data to PREZIES is not mandatory, data of hospitals implementing the bundle but choosing not to report the bundle to PREZIES were not available. From PREZIES data available mid-2015, we selected data from 2009 to 2014. Additionally, we only included the index surgeries, i.e. the surgical procedures especially selected by the DHPSP to measure compliance with the bundle and impact of bundle compliance on SSI incidence (Table 2) [9].

According to Dutch legislation, written consent from each individual patient was not required because the data from the PREZIES network is anonymized and was gathered as a legal task of the National Institute for Public Health and the Environment.

### Statistical analyses

We used surveillance data collected from medical files (surgeries on patient level) and analysed the four bundle elements, both separately and combined as a bundle, investigating (i) reporting, (ii) compliance, and (iii) the effect of compliance on the SSI risk. By definition, reporting refers to marking the variable in the PREZIES database with either a ‘yes’ (compliant) or a ‘no’ (non-compliant). Compliance is defined as answering ‘yes’. Complete bundle compliance was defined as compliance with all four elements within one patient (i.e. four times ‘yes’).

**Fig 1:**
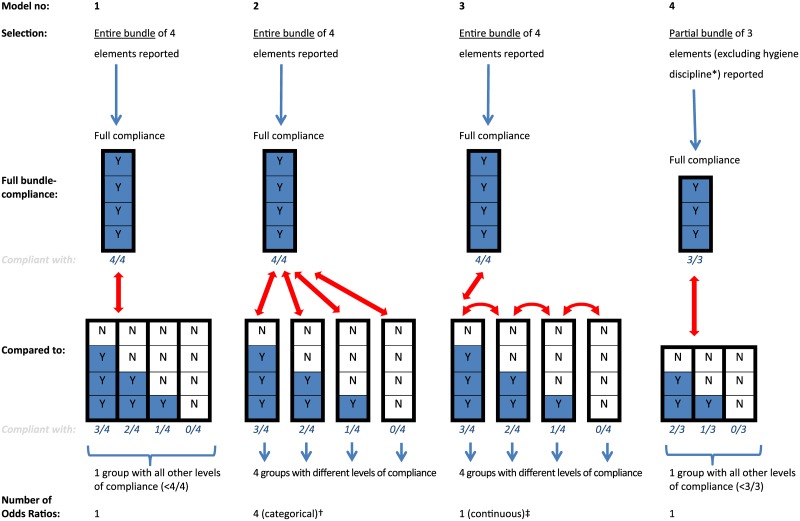
Statistical analysis: Four analyses comparing the influence of bundle-compliance on the SSI-risk. Comparisons made in Models 1 to 4 schematically represented. A stack of four boxes represents the entire bundle, a stack of three boxes represents the partial bundle. Compliance with a single bundle element is presented as a blue box with a Y (= Yes). Non-compliance with a bundle element is presented in a white box with an N (= No). Only bundles being completely reported were included (N = 62 486 for entire bundle, N = 99 371 for the partial bundle). Bundles with missing data concerning compliance were excluded. Key: * Model 4: The partial bundle does not include the bundle element hygiene discipline (‘minimising operating room door movements’), but is only composed of the bundle elements PAP-LG, hair removal and normothermia. † Model 2: Level of compliance entered as a *categorical* variable produces 4 Odds Ratios (ORs), comparing full compliance with the four other levels of compliance separately. ‡ Model 3: Level of compliance entered as a *continuous* variable produces 1 Odds ratio (OR), signifying the reduced risk for each single point increase in compliance-level.

1. To investigate bundle reporting, all data included was used. We calculated the reported numbers and percentages for each of the four elements separately and for the entire bundle; by year, by specialty and in total.
2. To calculate compliance with separate bundle elements, we selected patient surgeries with reported bundle data (without missing values), i.e. with a known status of (non-)compliance. We calculated compliance with each element as a percentage of the surgeries for which the element was reported.
3. We calculated the percentage compliance with the whole bundle using a selection of surgeries for which the entire bundle was reported. Both compliance with the four elements separately and with the bundle were plotted over time.
4. After that, we assessed the effect of bundle compliance on the risk of an SSI while selecting surgeries for which the entire bundle was reported. We used multivariate multilevel logistic regression techniques, creating Odds Ratios (ORs), to create five models; four for bundle compliance (Fig 1) and one for compliance with the separate bundle elements. To estimate the effect of bundle compliance, we calculated the level of compliance with the bundle as a number ranging from 0 to 4 for each individual with 0 referring to complete non-compliance (compliant with none of the four elements, 0/4) and 4 referring to full compliance (compliant with all 4 elements, 4/4). We then first (Model 1) compared the effect of full bundle compliance (4/4) with the effect of combined other levels of compliance (compliance levels 0 to 3, grouped) on the risk of an SSI. Secondly, we compared full compliance (4/4) with the other levels of compliance separately (3, 2, 1, or 0 out of 4). This analysis produced four estimates comparing each lower level of compliance with full compliance (compliance analysed as a categorical variable, Model 2) and one estimate signifying the relative risk caused by a single point increase in compliance level (compliance analysed as a continuous variable, Model 3). After that, we repeated Model 1 but this time with a partial bundle made up of the three evidence-based elements only (referred to as ‘partial bundle’ from this point onwards), not incorporating the expert-opinion based element of hygiene discipline (minimising door movements, Model 4). In this analysis we compared the effect of full compliance with the partial bundle (3/3) with all other levels of compliance combined (<3/3). Finally, to estimate the effect of compliance with individual bundle elements, we modelled the four bundle elements separately (Model 5).
5. Finally, as part of a sensitivity analysis we used the same statistical techniques to compare the SSI risk of surgeries with full bundle compliance (among bundles entirely reported) to the SSI risk of surgeries having no bundle reporting or having bundles not entirely reported in the PREZIES database.

All regression analyses were performed for all the medical specialties combined, while taking into account different baseline-risks between medical specialties (fixed effect) and differences between medical partnerships, between hospitals, and slopes (random effects). To correct for possible confounding by duration of participation to the surveillance (‘surveillance effect’) we checked year of surgery and duration of participation to the DHPSP at the time of surgery for their impact on the relationship between compliance and risk of infection. Furthermore, from the other variables available from the surveillance data, we considered the NNIS risk index, duration of surgery, ASA-score, wound class, having a prior surgery, multiple operations and having subsequent surgery to be potential confounders. We used forward selection, adding variables to the model if they changed the OR by 10% or more. For the individual bundle elements, we corrected for compliance with each of the other three bundle elements (making a model combining the four elements).

To validate interpretation of the ORs, we compared crude ORs with crude RRs (Risk Ratios) calculated using generalised linear models. All analyses were performed using the SAS 9.3 statistical package.

## Results

### Study inclusion and descriptives

Data on 232 274 surgeries performed from 2009 to 2014 were available for the 13 index surgeries of the DHPSP. However, because of the mandatory follow-up period of one year, the data from 2014 were incomplete at the time of analysis. We excluded cardiovascular surgeries (CAR, n = 13 736) because for this specialty it was impossible to meet the primary outcome (bundle compliance) as the bundle element hygiene discipline was not implemented for this specialty. We also excluded surgeries which were performed in the only hospital not participating in the DHPSP (n = 1 033), and 16 surgeries on patients who were younger than a year. A total number of 217 489 surgical procedures remained to be included. Distribution over the six remaining specialties and other baseline descriptives are presented in Table 3, which also includes the cumulative incidences per specialty.

**Table 3:** Baseline characteristics of surgeries with bundles not reported, partially reported, entirely reported but not fully compliant, and entirely reported with full compliance.

		Surgeries included in the study		Bundles not entirely reported N = 155 003 (71%)				Bundles entirely reported N = 62 486 (29%)			
				Not reported		Partially reported		Not entirely compliant		Full compliance	
		(N = 217 489)		N = 67 900 (31%)		N = 87 103 (40%)		N = 31 633 (15%)		N = 30 853 (14%)	
**DESCRIPTIVES**											
		**%**	**(N)**	**%**	**(N)**	**%**	**(N)**	**%**	**(N)**	**%**	**(N)**
Specialty*	**ORT**	**60**	(131 135)	**68**	(45 877)	**58**	(50 282)	**44**	(13 974)	**68**	(21 002)
	**BRE**	**7**	(14 310)	**7**	(4 592)	**7**	(6 025)	**7**	(2 057)	**5**	(1 636)
	**GIS**	**13**	(29 263)	**9**	(6 117)	**14**	(12 524)	**20**	(6 370)	**14**	(4 252)
	**GYN**	**17**	(36 158)	**15**	(10 171)	**16**	(14 137)	**26**	(8 226)	**12**	(3 624)
	**NEU**	**2**	(3 480)	**0.7**	(481)	**3**	(2 278)	**2**	(592)	**0.4**	(129)
	**VAS**	**1**	(3 143)	**1**	(662)	**2**	(1 857)	**1**	(414)	**0.6**	(210)
**Sex**	**Female**	**73**	(159 740)	**74**	(49 929)	**73**	(63 506)	**76**	(24 136)	**72**	(22 169)
**ASA score**	**1**	**28**	(60 679)	**26**	(17 791)	**28**	(24 558)	**31**	(9 757)	**28**	(8 573)
	**2**	**57**	(124 668)	**60**	(40 459)	**56**	(48 766)	**54**	(16 948)	**60**	(18 495)
	**3**	**12**	(25 039)	**12**	(7 912)	**12**	(10 412)	**10**	(3 152)	**12**	(3 563)
	**4**	**0.4**	(916)	**0.4**	(274)	**0.5**	(432)	**0.5**	(152)	**0.2**	(58)
	**5**	**0.1**	(125)	**<0.1**	(27)	**0.1**	(68)	**0.1**	(28)	**<0.1**	(2)
	**unknown**	**3**	(6 062)	**2**	(1 437)	**3**	(2 867)	**5**	(1 596)	**0.5**	(162)
**Wound class**	**1**	**82**	(179 048)	**87**	(59 220)	**81**	(70 675)	**74**	(23 369)	**84**	(25 784)
	**2**	**15**	(32 301)	**11**	(7 138)	**16**	(13 651)	**22**	(6 894)	**15**	(4 618)
	**3**	**0.9**	(2 024)	**0.7**	(475)	**1**	(939)	**1**	(416)	**0.6**	(194)
	**4**	**0.5**	(1 038)	**0.4**	(302)	**0.6**	(510)	**0.6**	(186)	**0.1**	(40)
	**unknown**	**1**	(3 078)	**1**	(765)	**2**	(1 328)	**2**	(768)	**0.7**	(217)
**NNIS risk**	**0**	**60**	(131 165)	**61**	(41 548)	**57**	(49 865)	**61**	(19 209)	**67**	(20 543)
**index**	**1**	**32**	(69 092)	**32**	(21 485)	**34**	(29 527)	**29**	(9 171)	**29**	(8 909)
	**2**	**4**	(8 232)	**4**	(2 637)	**4**	(3 528)	**3**	(1 049)	**3**	(1 018)
	**3**	**0.1**	(222)	**0.1**	(54)	**0.1**	(111)	**0.1**	(47)	**<0.1**	(10)
	**missing**	**4**	(8 778)	**3**	(2 176)	**5**	(4 072)	**7**	(2 157)	**1**	(373)
		**Med**	**(IQR)**	**Med**	**(IQR)**	**Med**	**(IQR)**	**Med**	**(IQR)**	**Med**	**(IQR)**
**Age (years)**		**65**	(24)	**66**	(21)	**66**	(24)	**62**	(34)	**66**	(21)
**Duration surgery (minutes)**		**67**	(41)	**68**	(40)	**69**	(41)	**60**	(42)	**65**	(36)
**Duration participation**†		**1.3**	(2.4)	**0.0**	(0.5)	**1.9**	(1.9)	**1.6**	(2.0)	**2.6**	(2.5)
**OUTCOME**											
	**Specialty**	**%**	**(N)**	**%**	**(N)**	**%**	**(N)**	**%**	**(N)**	**%**	**(N)**
**SSI incidence**	**ORT**	**1.4**	(1 855)	**1.5**	(666)	**1.4**	(709)	**1.5**	(209)	**1.3**	(271)
	**BRE**	**4.2**	(602)	**4.6**	(211)	**4.2**	(253)	**3.9**	(80)	**3.6**	(58)
	**GIS**	**6.6**	(1 919)	**8.1**	(497)	**7.3**	(911)	**5.1**	(324)	**4.4**	(187)
	**GYN**	**1.2**	(442)	**1.4**	(145)	**1.3**	(180)	**1.1**	(87)	**0.8**	(30)
	**NEU**	**0.8**	(28)	**0.8**	(4)	**0.9**	(21)	**0.5**	(3)	**0**	(0)
	**VAS**	**3.2**	(102)	**2.3**	(15)	**3.6**	(67)	**3.6**	(15)	**2.4**	(5)

Numbers presented are % (N) for categorical variables, and Median (IQR) for continuous variables. SSI incidences are presented as % (N) per specialty. Bundle reporting reflects reporting of (non-)compliance with the bundle in the PREZIES database: not reported, partially reported or entirely reported. Complete reporting of the bundle (n = 62 486) refers to all four elements of the bundle being reported (either compliant or non-compliant, no missing values). Full bundle compliance (N = 30 853) refers to all four elements of the bundle being compliant. Not entirely compliant refers to no compliance or partial compliance with the bundle.

* Specialties: ORT = Orthopaedic Surgery, GYN = Gynaecology, GIS = Gastro Intestinal Surgery, BRE = Breast Surgery, NEU = Neurosurgery, VAS = Vascular Surgery

^†^ Duration participation: The duration of the hospital’s participation in the DHPSP at the time of surgeryOther abbreviations: ASA score = American Society of Anesthesiologists physical status classification system, DHPDP = Dutch Hospital Patient Safety Program, IQR = Inter Quartile range, Med = median, NNIS risk index = National Nosocomial Infections Surveillance risk index, SSI = Surgical Site Infection (deep and superficial SSIs combined)

### Reporting

In the PREZIES database, 62 486 (29%) of the surgical procedures had complete reporting of the entire bundle (i.e. all four elements reported compliant/non-compliant, no missing values). Of the remaining 155 003 (71%) surgeries, 87 103 (40%) had partial reporting of the bundle and for 67 900 (31%) surgeries the bundle was not reported in the PREZIES database. Complete reporting of the partial bundle excluding hygiene discipline was 46% (n = 99 371). Reporting of (non-)compliance with the bundle elements varied between the elements. It increased over time for each element, but was lowest for hygiene discipline (Table 4). Reporting of the entire bundle logically also increased over time, but was still below 50% in 2013 and 2014.

**Table 4:** Reporting of and compliance with the bundle and its elements.

	2009		2010		2011		2012		2013		2014		TOTALS		
	(N = 31 318)		(N = 44 110)		(N = 52 222)		(N = 44 110)		(N = 36 881)		(N = 8 918)		N	N	N
	%	(N)	%	(N)	%	(N)	%	(N)	%	(N)	%	(N)			
**A) Reporting of bundle elements (N = 217 489)**													**Reported**	**Missing**	**TOTAL**
PAP-LG*	**28**	(8 763)	**55**	(24 172)	**65**	(33 971)	**80**	(35 109)	**80**	(29 363)	**87**	(7 720)	139 098	78 391	**217 489**
**Hair removal**	**17**	(5 429)	**51**	(22 418)	**60**	(31 539)	**78**	(34 234)	**80**	(29 255)	**87**	(7 747)	130 622	86 867	**217 489**
**Normothermia**	**16**	(5 119)	**47**	(20 614)	**53**	(27 511)	**64**	(28 093)	**68**	(24 902)	**67**	(5 976)	112 215	105 274	**217 489**
**Hygiene discipline**	**13**	(3 945)	**28**	(12 297)	**30**	(15 607)	**42**	(18 726)	**48**	(17 795)	**46**	(4 087)	72 457	145 032	**217 489**
**B) Reporting of bundle (N = 217 489)**													**Entirely reported**	**Not entirely reported**†	**TOTAL**
**ENTIRE BUNDLE**	**10**	(3 097)	**23**	(10 136)	**27**	(14 263)	**37**	(16 426)	**45**	(16 625)	**42**	(3 739)	**62 486**	155 003	**217 489**
**C) Compliance with bundle elements (N = 217 489)**													**Compliant**	**Non-compliant**	**TOTAL**
PAP-LG*	**66**	(5 810)	**70**	(16 921)	**72**	(24 302)	**79**	(27 610)	**81**	(23 848)	**85**	(6 561)	105 052	34 046	139 098
**Hair removal**	**72**	(3 924)	**83**	(18 661)	**89**	(28 144)	**96**	(32 738)	**96**	(28 159)	**96**	(7 454)	119 080	11 542	130 622
**Normothermia**	**43**	(2 189)	**61**	(12 591)	**78**	(21 462)	**84**	(23 619)	**88**	(21 890)	**89**	(5 294)	87 045	25 170	112 215
**Hygiene discipline**	**39**	(1 554)	**54**	(6 631)	**73**	(11 389)	**84**	(15 750)	**89**	(15 791)	**82**	(3 369)	54 484	17 973	72 457
**D) Compliance with bundle (N = 62 486)**													**Compliant**	**Not entirely compliant**‡	**TOTAL**
**ENTIRE BUNDLE**	**19**	(584)	**24**	(2 450)	**34**	(4 882)	**56**	(9 233)	**68**	(11 330)	**63**	(2 374)	30 853	31 633	**62 486**

Numbers displayed are numbers of bundle elements and numbers of entire bundles being reported (i.e. compliant or non-compliant), or being compliant. Numbers are presented by year and in total, and are % (N) unless stated otherwise. Reporting percentages and compliance percentages are calculated using different denominators.

(A) Percentage reported bundle elements = N reported bundle elements/N bundle elements total included per year (denominator taken from header row).

(B) Percentage reported bundles = N reported bundles/N bundles total included per year (denominator taken from header row).

(C) Percentage compliance bundle element = N compliant elements/N reported elements per year (denominator taken from Part A of the table).

(D) Percentage compliance bundle = N compliant bundles/N reported bundles per year (denominator taken from Part B of the table).

Numbers presented are % (N) unless stated otherwise.

*PAP-LG = Peri-operative Antibiotic Prophylaxis according to Local Guidelines

^†^ Bundle reporting: Not entirely reported = bundle is not reported or partial reported

^‡^ Bundle compliance: Not entirely compliant = no compliance or partial compliance with the bundle

Data from 2014 are incomplete because of the mandatory delay in follow-up of one year, which caused a delay in reporting the data.

### Compliance

**Fig 2:**
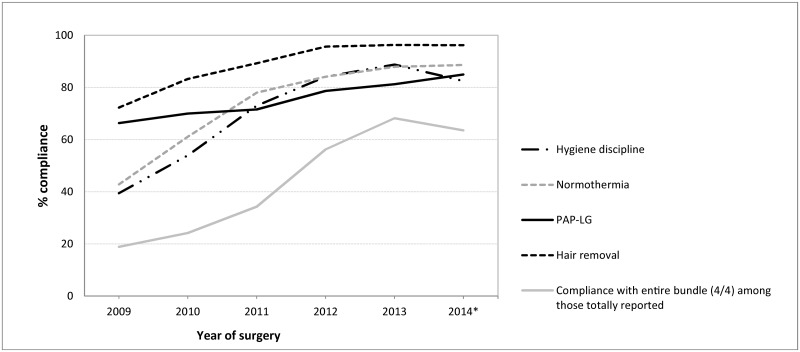
Compliance over time. Compliance with the bundle elements and with the bundle over time. Compliance with each bundle element was calculated using surgeries for which the element was reported, i.e. not missing (n = 72 457 for hygiene discipline, n = 112 215 for normothermia, n = 130 622 for hair removal, and n = 139 089 for PAP-LG). Bundle compliance was calculated within the group of surgeries for which (non-)compliance was reported for the entire bundle (n = 62 486). * Data from 2014 are incomplete because of the mandatory delay in follow-up of one year, which caused a delay in reporting the data. PAP-LG = Peri-operative Antibiotic Prophylaxis according to Local Guidelines.

Bundle compliance was calculated within the group of surgeries with complete bundle reporting (n = 62 486). Compliance with a bundle element was calculated using surgeries for which the element was reported (varying from n = 72 457 for hygiene discipline to n = 139 089 for PAP-LG, Table 4). Compliance with each of the bundle elements and with the bundle increased and stabilised over time (Table 4, Fig 2). Compliance with the elements ranged from 39% to 72% in 2009 but, by 2014, had reached 82% to 96%. Bundle compliance increased over time from less than 20% to 64%.

### Effect of bundle compliance on SSI risk

The results in Table 5A and 5B (models 1, 2 and 4) show that the risk of an SSI was lower for surgeries with full compliance compared to surgeries with lower compliance levels. Crude ORs were all statistically significant below 1 (range 0.73–0.87) despite relatively broad 95% CIs. The final multilevel ORs either remained similar or slightly decreased (range 0.63–0.86, indicating a risk reduction of 14% to 37%), keeping significance. The OR of 0.87 (95%CI 0.81 to 0.94) for a single point increase in compliance level (model 3) indicates that one point increase of the compliance level reduced the risk of an SSI by 13%.

**Table 5:** Bundle compliance and compliance with individual bundle elements: Odds ratios (ORs) for the risk of an SSI.

Model No.	Effect of:	Compared to compliance with:	ORs for the risk of an SSI			Multilevel, corrected		Corrected for:
			Crude	Multilevel				
			OR	OR	95%CI	OR	95%CI	
**A) Bundle compliance, entire bundle (4 elements)**								
**1**	Complete compliance (4/4)	<4 elements, grouped	0.78	0.76	(0.64 to 0.92)	-	-	-
**2**	Complete compliance (4/4)	0 elements	0.73	0.63	(0.40 to 0.99)	-	-	-
		1 elements	0.78	0.69	(0.49 to 0.97)	-	-	-
		2 elements	0.73	0.78	(0.61 to 0.997)	-	-	-
		3 elements	0.82	0.82	(0.68 to 0.997)	-	-	-
**3**	Each point increase in compliance level		0.91	0.87	(0.81 to 0.94)	-	-	-
**B) Bundle compliance, partial bundle (3 elements)**								
**4**	Complete compliance (3/3)	<3 elements, grouped	0.87	0.86	(0.76 to 0.98)	-	-	-
**C) Compliance with individual bundle elements**								
**5**	Compliance with:	PAP-LG	0.84	0.96	(0.82 to 1.12)	1.02	(0.87 to 1.20)	HR, N, HD
		Hair removal	0.87*	0.68	(0.55 to 0.86)	0.76	(0.60 to 0.96)	P, N, HD
		Normothermia	0.88	0.83	(0.72 to 0.96)	0.89	(0.76 to 1.04)	P, HR, HD
		Hygiene discipline	0.77	0.74	(0.62 to 0.87)	0.79	(0.66 to 0.93)	P, HR, N

Effect of compliance with the bundle (models 1 to 4) and of compliance with the separate bundle elements (model 5) on SSI risk. Analyses for models 1, 2, 3 and 5 are based on 62 486 surgeries (entire bundle) and for model 4 on 99 371 surgeries (partial bundle, i.e. bundle excluding hygiene discipline). All multilevel ORs take into account the different baseline-risks between medical specialties (fixed effect), and differences between medical partnerships and slopes (random effects). Crude ORs were similar to crude RRs (differences all <0.01), implying that the ORs may be interpreted as RRs.

For models 1 to 4 no confounders were detected. For model 5 no slopes were detected, but the effect of compliance with each element was corrected for presence of compliance with the other three elements.

* Not significant

HR = Hair removal, N-Normothermia, P = PAP-LG, HD = Hygiene discipline (door movements)

Multilevel ORs for compliance with the individual elements were statistically significant below 1 for hair removal, normothermia and hygiene discipline (0.68, 0.83 and 0.74 respectively, Table 5C, model 5). The OR for PAP-LG (following local guidelines for PAP), however, was not significantly reduced (0.96). After correcting for confounding by the other elements, all ORs increased but remained significantly reduced for hair removal and hygiene discipline. Not enough data was available to reliably analyse the individual elements for each medical specialty separately. However, a limited number of additional analyses could be performed which indicated that compliance with the element of hair removal significantly reduced the risk of an SSI for breast surgeries (OR 0.52, 95%CI 0.28 to 0.98), while hygiene discipline was associated with a reduced OR for gastro-intestinal surgery (OR = 0.71, 95%CI 0.55 to 0.91). No other associations were found (results not presented).

Comparing the group with complete reporting (29%, n = 62 486) to the remaining surgeries without (complete) bundle reporting (71%, n = 155 003), the results in Table 3 suggest that the SSI incidences were mostly higher for this latter group. Sensitivity analyses comparing surgeries with full bundle compliance (n = 30 853) to surgeries with partial bundle reporting (n = 87 103) or with no bundle reporting to PREZIES (n = 67 900), confirmed this. While the risk reduction of full compliance compared to incomplete compliance was 24% (model 1), it was 34% when compared to partially reported bundle data (multilevel OR 0.66, 95%CI 0.56 to 0.79) and 25% when compared to surgeries without bundle data reported to PREZIES (multilevel OR 0.75, 95%CI 0.71 to 0.92, corrected for NNIS risk index). Note that the latter group is a heterogeneous group consisting of hospitals implementing the bundle but not reporting on national level, as well as hospitals not implementing the bundle at all.

For all the analyses performed, the crude ORs were similar to crude RRs (differences all <0.01, results not presented), implying that the ORs may be interpreted as RRs.

## Discussion

### Key findings

We observed that compliance with the entire bundle of care, but also compliance with a part of the bundle, significantly reduced the risk of an SSI. Full compliance compared to other compliance-levels resulted in a risk reduction varying from 14% to 37%, depending on the group that was used as a comparison. Thus, it is possible to reduce the cumulative incidence of SSIs by defining and adhering to best practices.

### Main results

It took considerable time for the hospitals to implement the bundle, and hospital personnel considered the workload of reporting the bundle and handling feedback to be high. This is in line with our findings that for more than 70% of the included surgeries, the bundle was not, or was not completely, reported in the PREZIES database. PAP-LG was most frequently reported by the hospitals, but the other three bundle elements had considerably lower reporting levels. Nevertheless, it is the bundle approach, and not compliance with separate interventions that is claimed to be successful, as the bundle approach is considered to ensure the consistent application of all measures [9, 13]. Our findings of a stepwise increase in the protective effect of full bundle compliance (from 18% (0.82) to 37% (0.63)) when the level of compliance to which it was compared decreased (from 3 to 0), and a 13% risk reduction additionally demonstrated for each point increase in compliance, support this claim. As compliance increased over time, the protective effect of compliance could theoretically have been biased by the effect of duration of participation to the surveillance (‘surveillance effect’), but both year of surgery and duration of participation in the DHPSP (as proxy for duration of participation to the surveillance) proved to be no confounders.

Many professionals’ doubts about the validity of using operating room door movements as a surrogate for hygiene discipline may have contributed to the lower reporting of this element. Nevertheless, we detected a greater risk reduction when the complete bundle (24%) was adhered to, compared to the partial bundle (14%), suggesting that paying attention to door movements as an approximation for hygiene discipline further reduced the risk of an SSI. The SSI risk of 0.79 for compliance with the element hygiene discipline, confirmed this. As this result was most evident in gastro-intestinal surgery (i.e. contaminated area), it is likely that minimising the number of door movements improved the degree of attention given to all tasks in the operating rooms and thus created a more attentive and accurate work environment [19], thereby reducing spill of contaminated material.

Compliance with ‘no unnecessary hair removal’ was also associated with a risk reduction in our study. This protective effect was highest for breast surgeries, where surgical incisions often involve the armpit. Compliance with normothermia and with following local guidelines for antibiotic prophylaxes (PAP-LG), however, did not significantly reduce the SSI risk. The lack of effect of normothermia is probably due to a lack of power, but the lack of effect of PAP-LG may be explained by the definition of PAP-LG. First, PAP-LG was defined as a multi-part question, which is difficult by nature to report. Secondly, not exactly following the local guidelines for PAP did not necessarily mean that no PAP was given at all. If local guidelines were different from the national guidelines, providing PAP according to national guidelines resulted in being non-compliant with the local guidelines. Also, small deviations in PAP administration (for instance timing) might have led to it being considered non-compliant. The actual effect of such deviations on the risk of an SSI might have been low, and thus results found for PAP-LG in this study do not imply that administration of PAP itself (which is advocated by several SSI prevention guidelines and the WHO) [11, 20, 21] is not useful.

### Comparison with literature

To the best of our knowledge, this is the first European study analysing the effect of adherence to an SSI prevention bundle using case-based data from a national database which includes several types of surgery.

Previous European and non-European studies evaluated the effect of a surgical care bundle on SSI incidences [13, 22–40]. The bundles studied were different, but most included elements such as appropriate dose of PAP, appropriate timing of PAP, discontinuation of PAP, or hair removal [13]. Most studies, however, focused on only one type of surgery [23, 25–32, 34, 35, 38–40], or investigated the effect of a bundle using a before-after design (without measuring compliance, or without directly incorporating compliance into the analyses) [23, 25, 27–35, 37, 39, 40]. A study similar to ours was performed in the USA in 2011 and included over 60 000 surgeries among 5 medical specialties [22]. The researchers did not find a significant effect of bundle compliance but may have underestimated the effect of compliance as they adjusted for all variables known to predict SSIs (which probably are not all confounders). By doing this, they predicted the overall odds of an SSI rather than estimating the odds of an SSI for bundle compliance [41]. Also, with similar numbers to analyse, we noticed that adjusting for many variables caused our models to become unstable and unreliable because of the low numbers in subgroups.

Summarised, most previous studies found that implementing a bundle coincided with decreased SSI incidence [23, 25, 27–31, 33, 34, 37–39], or found no significant effect [24, 32, 35, 37] probably due to lack of power [13]. Therefore, in 2015, Tanner et al published a meta-analysis of the effect of surgical care bundles on the risk of an SSI for colorectal surgery [13] and included 8 515 surgeries from 13 of the studies mentioned above. Comparing SSI-incidences before and after the introduction of a bundle, they found that the use of a bundle reduced the risk of an SSI by 45% (Risk Ratio 0.55). They did not, however, publish any information about compliance rates.

### Strengths and limitations

This study is a valuable addition to the studies mentioned above, because it included almost 30 000 gastro-intestinal tract surgeries of which 10 622 had complete reporting of compliance data for the entire bundle, and 16 922 for the partial bundle. In addition, this study enables more generalisations to be made, because we included several types of surgery, and presented and used compliance data. Measuring and aiming to minimize door movements in order to achieve hygiene discipline is unique to our study and has been subject to debate due to the lack of evidence for its effectiveness. However, the additional risk reduction of 10–21% found in our study should add to the current body of evidence supporting the importance of raising awareness in the operating room. The conclusions regarding PAP-LG on the other hand are, due to the unusual definition of this bundle element, not generalisable to differently formulated PAP bundle elements and probably also not to other countries. Numbers included in our study were relatively high, but retaining robust models was still challenging.

As only surgeries with a known status of bundle-compliance could be used to compare the SSI risk of full compliance with that of not being fully compliant, only surgeries with complete bundle reporting were selected for this comparison. Of all included surgeries, only 29% (62 486) had complete bundle reporting. Although this percentage seems low, in this setting without mandatory reporting it is in line with the expectations, and is even higher than the percentage reporting found in a recent Dutch randomised trial investigating the use of antibiotic checklists [42]. In fact, another 40% of the surgeries (n = 87 103) had partial bundle reporting, and part of the group without bundle reporting to PREZIES (31%, n = 67 900) consisted of surgeries from hospitals that did implement the bundle but choose to report (non-)compliance only to their own staff (internal reporting). As such, the latter group is a heterogeneous group consisting of hospitals implementing the bundle but not reporting on national level, as well as hospitals not implementing the bundle at all. Therefore, the estimated 25% risk reduction for this group probably underestimates the effect to be expected when surgeries with reported full compliance had been compared to surgeries from hospitals were no effort was taken to implement the bundle. These results indicate that putting effort in full bundle reporting by itself may already reduce SSI incidence, and hence the results found in this study (i.e. impact of compliance among bundles reported) are most probably an underestimation of the actual effect of introducing and using a bundle of care.

Because of the small numbers of SSIs per medical specialty, the study was underpowered to stratify by medical specialty. However, adding medical specialty to the model as a confounder yielded robust and reliable estimates. Using models 1 and 4, other potential confounders were adequately tested for their influence on the SSI risk. These covariates, however, did not confound the relationship between bundle compliance and SSI risk. They were therefore not added and were for this reason also not considered for the other models. Finally, log binomial regression analyses to calculate RRs did not produce results, forcing us to calculate ORs using logistic regression techniques. However, comparison of crude ORs and RRs revealed that, in this study, the ORs can be interpreted as RRs.

### Implications and conclusion

The elements included in our bundle are slightly different from those used in other studies, but in general it is the bundle approach that is believed to create awareness and to improve infection prevention [9, 13]. The results of our study confirm that bundle compliance reduces the risk of an SSI. In fact, as reporting of, and compliance with, the bundle can still be improved, an even greater effect might be achieved. It costs, however, a lot of effort to continuously report bundles and to handle feedback, especially if bundle reporting is not fully automated. Whether, and for how long any positive effect of reporting bundle compliance endures after successful implementation is also unknown at this point. An investigation should be made into whether the periodic measurement of bundle compliance, with adequate feedback, could also maintain awareness, as this would substantially reduce the workload.
